# Expression pattern of human ATP-binding cassette transporters in skin

**DOI:** 10.1002/prp2.5

**Published:** 2013-09-08

**Authors:** Saya Takenaka, Tomoo Itoh, Ryoichi Fujiwara

**Affiliations:** School of Pharmacy, Kitasato University5-9-1 Shirokane, Minato-ku, Tokyo, 108-8641, Japan

**Keywords:** ABC transporter, cancer, mRNA expression, skin, toxicology

## Abstract

ATP-binding cassette (ABC) transporters transport a variety of substrates across cellular membranes coupled with hydrolysis of ATP. Currently 49 ABC transporters consisting of seven subfamilies, ABCA, ABCB, ABCC, ABCD, ABCE, ABCF, and ABCG, have been identified in humans and they are extensively expressed in various tissues. Skin can develop a number of drug-induced toxicities' such as Stevens–Johnson syndrome and psoriasis. Concentration of drugs in the skin cells is associated with the development of adverse drug reactions. ABC transporters play important roles in absorption and disposition of drugs in the cells; however, the expression pattern of human ABC transporters in the skin has not been determined. In this study, the expression patterns of 48 human ABC transporters were determined in the human skin as well as in the liver and small intestine. Most of the ABCA, ABCB, ABCC, ABCD, ABCE, and ABCF family members were highly or moderately expressed in the skin, while ABCG family members were slightly expressed in the skin. Significant interindividual variability was also observed in the expression levels of those ATP transporters in the skin, except for ABCA5 and ABCF1, which were found to be expressed in all of the human skin samples tested in this study. In conclusion, this is the first study to identify the expression pattern of the whole human ABC family of transporters in the skin. The interindividual variability in the expression levels of ABC transporters in the human skin might be associated with drug-induced skin diseases.

## Introduction

Most of the ATP-binding cassette (ABC) transporter genes encode functional transmembrane proteins that utilize the energy of ATP hydrolysis to transport their substrates across membranes (Dean et al. 2001a). ABC transporters transport a wide variety of endogenous compounds across extra- and intracellular membranes, including cholesterol, peptides, iron, ions, bile salts, and lipids (Borst et al. 2000; Schmitz et al. 2000, 2001; Lu et al. 2001). They also transport exogenous compounds such as clinically used drugs (Glavinas et al. 2004). Currently 49 ABC transporter subtypes, including a pseudogene, have been identified in humans, and they are divided into seven subfamilies, ABCA, ABCB, ABCC, ABCD, ABCE, ABCF, and ABCG (Dean et al. 2001a). The ABCA family forms the second largest gene family, consisting of 12 subtypes (Broccardo et al. 1999). Evolutional analysis has revealed a gene cluster, which locates on chromosome 17q24, encoding ABCA5, ABCA6, ABCA8, ABCA9, and ABCA10 (Arnould et al. 2001), while most other ABC transporter genes are dispersed in the mammalian genome. ABC transporters are expressed in various tissues such as the liver, intestine, kidney, and brain (Langmann et al. 2003). In the small intestine, certain ABC transporters mediate active efflux of drugs, playing an important role in poor absorption and low bioavailability of drugs (Kato et al. 2009). Certain subtypes of the ABC transporters, such as ABCB1 and ABCB4, are involved in multidrug resistance (MDR), as they decrease drug concentration in multidrug resistant cancer cells by exporting drugs to outside the cell (Rappa et al. 1997; Siddiqui et al. 2003).

The human ABCA transporter subfamily consists of 12 members (Arnould et al. 2001). Several ABCA family transporters are linked to genetic diseases such as ABCA1 to Tangier disease and high-density lipoprotein deficiency and ABCA4 to Stargardt disease (Dean et al. 2001b). The human ABCB transporter subfamily consists of 11 members. One of the ABCB family transporters, ABCB1, is known as a MDR1 protein, which is also known as permeability glycoprotein (P-gp), as the expression of ABCB1 in the cells develops resistance to anticancer drugs (Siddiqui et al. 2003). While ABCB4 is another member of MDR proteins, other ABCB family transporters have different roles as ABCB2 and ABCB3 are called antigen peptide transporter (TAP) 1 and TAP2, respectively (Dean et al. 2001a). ABCB11 is known as a bile salt export pump (BSEP) and it is responsible for bile acid-dependent bile flow at the apical membrane of hepatocytes (Childs et al. 1995). The ABCC family forms the largest gene family, consisting of 13 subtypes. Except for ABCC13, which is a pseudogene, the other 12 ABCC subfamily genes encode functional proteins such as ABCC1 and ABCC2, which are known as multidrug resistance-associated protein (MRP) 1 and MRP2, respectively. Similar to MDR proteins, MRP plays an important role in MDR (Szakács et al. 2006). The proteins encoded by ABCC1, ABCC2, ABCC3, ABCC4, ABCC5, ABCC6, ABCC10, ABCC11, and ABCC12 are the MRP members called MRP1 to MRP9, respectively. Several ABCC transporters are linked to genetic diseases such as ABCC2 to Dubin–Johnson syndrome and ABCC7 (cystic fibrosis transmembrane conductance regulator) to cystic fibrosis (Dean et al. 2001b). The ABCD subfamily contains four genes that encode half-transporters expressed exclusively in the peroxisome. One of the ABCD members, ABCD1, is linked to a genetic disease, adrenoleukodystrophy (Dean et al. 2001b). The ABCE and ABCF subfamilies are composed of genes that have ATP-binding domains that are closely related to those of the other ABC transporters. However, these genes do not encode any transmembrane domains (Dean et al. 2001a). The ABCG family transporters consist of half-transporters, which form an oligomer to form the functional transporter. ABCG1, ABCG4, ABCG5, and ABCG8 are involved in the ATP-dependent translocation of steroids and lipids, while ABCG2 has been identified as a multidrug transporter that confers resistance on cancer cells. Several ABCG transporters are linked to genetic diseases such as ABCG5 and ABCG8 to sitosterolemia (Dean et al. 2001b).

The expression pattern of ABC transporters in various human tissues was examined previously (Kaminski et al. 2001; Langmann et al. 2003; Ohtsuki et al. 2007), revealing that most of the ABC transporters were expressed not only in the liver and small intestine but also in organs that are mainly involved in secretory function (adrenal gland), barrier function (lung, trachea), and tropic function (placenta, uterus). However, little is known about the expression pattern of human ABC transporters in the skin. In this study, the expression patterns of 48 human ABC transporters were determined in the human skin as well as in the liver and small intestine.

## Materials and Methods

### Chemicals and reagents

The human total skin, liver, and small intestine RNA was purchased from Origene Technologies (Rockville, MD), Agilent Technologies (Santa Clara, CA), and Applied StemCell (Sunnyvale, CA). The total skin RNA from malignant melanoma patients was purchased from Origene Technologies. Primers were commercially synthesized at Invitrogen (Carlsbad, CA). Ex Taq DNA polymerase was purchased from Takara Bio (Shiga, Japan). All other chemicals and solvents were of analytical grade or the highest grade commercially available.

### Semi-quantitative and quantitative reverse transcription polymerase chain reaction

The complementary DNA (cDNA) was synthesized from total RNA using ReverTra Ace (TOYOBO, Osaka, Japan) according to the manufacturer's protocol. The reverse-transcribed mixture was diluted 10-fold and a 1-μL portion of the diluted solution was added to polymerase chain reaction (PCR) mixtures (25 μL). The amplification was performed by denaturation at 98°C for 10 sec, annealing at 60°C for 30 sec, and extension at 72°C for 30 sec for 40 cycles with Ex Taq DNA polymerase. The sequences of primers used in this study are summarized in Table 1. The PCR products (20 μL) were analyzed by electrophoresis with 2% agarose gel and visualized by ethidium bromide staining. Expression of glyceraldehyde-3-phosphate dehydrogenase (GAPDH) mRNA was used as an internal control for the cDNA quantity and quality. Quantitative reverse transcription PCR (Q-RT-PCR) was performed with SYBR Premix Ex Taq II (Takara), and the reactions were run in a CFX96™ Real-Time PCR Detection System (BioRad, Hercules, CA).

**Table 1:** Primer sequences for human ABC transporters

Subtypes	Forward primers (5′ to 3′)	Reverse primers (5′ to 3′)
ABCA1	TACATCTCCCTTCCCGAGCA	CAGCATCTTCCTCAGTGCCA
ABCA2	GTGGGCAACGTGACTCACTA	GCCGCGTTGTCAATGGTATC
ABCA3	TGCTGCTCAGCTTCACCTAC	GCCATGTTGGCTTTGCTGAA
ABCA4	CCCTCATGCAGAATGGTGGT	GCTGCAGGTGAATCAGGAGT
ABCA5	GACTGCAGGCCCATATCCTC	CTGTGGCCAAGTAAGGCAGT
ABCA6	TCCTGACCCTTCAGGAGACT	ACCCAGGATTGCCGTGATTT
ABCA7	CTTGCACAGCTTGTTGGAGG	GCTCCATCAGGTCACTAGCC
ABCA8	GGCCCTTTTCTTGGCACTTG	CAGGCCGGTGAGGAAAGATT
ABCA9	TGTTGCCCAAGGAGGAGTTG	ACCAAGCCCGTAAGGAAAGG
ABCA10	ACAGAACACTGTTGGGCCAT	ATCCCCAGGAGAGCCAGAAT
ABCA12	CTACTGCTGGATGAGCCGAG	CCTCGTCCAAACCTGCTCTT
ABCA13	TCACCGAGCCAGTTTACCAC	ACCCTGTTGCCACTGAACAA
ABCB1	CGTGGTTGGAAGCTAACCCT	TGCTGCCAAGACCTCTTCAG
ABCB2	CGTTGTCAGTTATGCAGCGG	ATAGATCCCGTCACCCACGA
ABCB3	GGCTGCTTCACCTACACCAT	GCATCCTGGATCTCCCGAAG
ABCB4	CATGGCCATAGCTCACGGAT	GCAGCAACAAGAACTCCAGC
ABCB5	GGTGAAGGGCTGGAAACTCA	CCTGGGCCCTAAAGGCTATG
ABCB6	GTTTGTCCTGGGTCTCTGGG	AAGGTGCCTTCTCAGTCAGC
ABCB7	CTGCTCGCGATGCATTCTTG	GGAATCTGCTGGTAGGCTCG
ABCB8	TGCTCCGACAAGACATCACC	CCTGACACTGGCGAGACAAT
ABCB9	TTGGTGTGGCCAAGAACAGT	TGTACGTCCACACGAACAGG
ABCB10	TGCGGTTGGATTTCTCACGA	CACACAGAAACACGGCACTG
ABCB11	GCCCTTTTCATTCAGCGCAT	TGCTTTGGCATAGGCCTTCA
ABCC1	AGGACACGTCGGAACAAGTC	GGAAGTAGGGCCCAAAGGTC
ABCC2	TCCTTTGCAAGTGACCGTGA	CCTTCCTGGCCAAGTTGGAT
ABCC3	CTGTGCACACAGAAAACCCG	GGACACCCAGGACCATCTTG
ABCC4	GAGTTGCAAGGGTTCTGGGA	AAAGTCAGCACCGTGGCATA
ABCC5	TCTGAAGCCCATCCGGACTA	CACGTCAGAAGACTCGTGCT
ABCC6	CACAGTTTGTGCTGTCCTGC	CCAAGCGACCAGAGGTCTTT
ABCC7	CCCAGCCATTTTTGGCCTTC	GACGCCTGTAACAACTCCCA
ABCC8	CTATGGGATGCTGCTCCTCG	GAGCCGTTGGTAGTTGGTGA
ABCC9	CCTCTTTATGCCAGCCGTGA	CTGTGATGCAGAAACGCAGG
ABCC10	CCTAGTGCTGACCGTGTTGT	TAGGTTGGCTGCAGTCTGTG
ABCC11	ACCCCGCTCATGATCCAAAG	ATGCCCAGAAGTGCATCGAA
ABCC12	TGACCCACATCTCTGTTGGC	CAGCCTGATGCAGGTCAGAA
ABCD1	GCCTATGGAGCCCACAAAGT	GCCACATACACCGACAGGAA
ABCD2	TGGCTTTGGCCTTCAGAACT	AGTCCTGCTAGTAGGGTGGG
ABCD3	CGCTGCCATGCCTCTTATCT	GAGCCTTACTCGGAAGCACA
ABCD4	GTTGGCTTGATCCCCAGTCA	CAGCACGTTGAGGGTGTAGT
ABCE1	GTTGCCTATCCCTCGTCCAG	TGTCCCCTTTGCAGCCTTAG
ABCF1	GCACTCAAGGGCAAAAAGGG	CACTTGGCGCTCATACTCCA
ABCF2	GCGGAGTGTGAGAAGCTCAT	TGGGAGACGAGGACCAAGAT
ABCF3	AAGCGCTCAGAGAAGGACAC	GCAACCTCTTGCTCAACGTG
ABCG1	GATGGTGTCGGCACATCTGA	GTGCAAATGATGGAGCGACC
ABCG2	TGATAAATGGAGCACCGCGA	GCCAGTTGTAGGCTCATCCA
ABCG4	ACCACATCACTGAAGCCCAG	AATGTAGACTTGCCAGCCCC
ABCG5	CATGTGGCAGACCGACTGAT	TTCAGGACAAGGGTAACCGC
ABCG8	GTGAGAAGTGGGCAGATGCT	TCCTCCACCCTTTTGTCACG

## Results

### Validation of the primers and PCR reactions

In this study, the expression of 12 ABCA, 11 ABCB, 12 ABCC, 4 ABCD, 1 ABCE, 3 ABCF, and 5 ABCG transporters was examined in the human skin as well as the liver and small intestine. Total RNA from human skin, the liver, and small intestine was from three, one, and one individual donors, respectively. Primers used in the study were established with a program, Primer Blast (National Institutes of Health), and we confirmed that all of the primer sets produced specific bands. All of the PCR reactions were carried out with the Ex Taq DNA polymerase (Takara).

### ABCA family transporter expression

**Figure 1:**
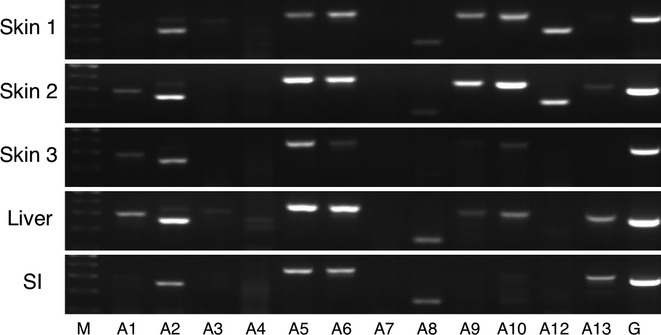
RT-PCR analysis of ABCA family transporter mRNA in human tissues. Total RNA samples of human skin, liver, and small intestine were analyzed by RT-PCR using primers specific for each ABCA subtypes. SI, small intestine; M, 100 base pair marker; G, GAPDH.

It was demonstrated that the expression level of ABCA5 was significantly high in the human skin and the high expression was observed in all of the three skin samples, while it was also highly expressed in the liver and small intestine (Fig. 1). The higher expression of ABCA2, ABCA6, ABCA9, ABCA10, and ABCA12 was observed in the skin; however, the interindividual variability in the expression level of these subtypes was also shown, as certain individual(s) slightly or barely expressed them in the skin. The expression of ABCA1, ABCA3, ABCA4, ABCA8, and ABCA13 was moderate or absent in the skin, liver, and intestine. In this study, it was shown that ABCA7 was hardly expressed in the examined tissues.

### ABCB family transporter expression

**Figure 2:**
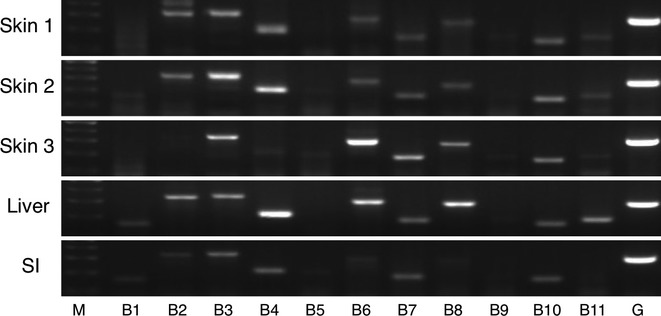
RT-PCR analysis of ABCB family transporter mRNA in human tissues. Total RNA samples of human skin, liver, and small intestine were analyzed by RT-PCR using primers specific for each ABCB subtypes. SI, small intestine; M, 100 base pair marker; G, GAPDH.

It was shown that ABCB3 was highly expressed in all of the three skin samples (Fig. 2). The higher expression of ABCB2, ABCB4, and ABCB6 was observed in the skin; however, there was the interindividual variability in these expressions. While it was moderate, ABCB10 was expressed equally in all of the skin samples, as well as in the liver and small intestine. Although the slight expression of ABCB7, ABCB8, and ABCB11 was observed in the skin, some of these expressions were not detected in certain individual(s), indicating the presence of the interindividual variability in the expression level of these subtypes. Expression of ABCB1, ABCB5, and ABCB9 was barely detected in the human skin. In the liver, most of these family members were mildly expressed except for ABCB5 and ABCB9. In the small intestine, the expression of ABCB1, ABCB2, ABCB3, ABCB4, ABCB5, ABCB6, ABCB7, and ABCB10 was detected.

### ABCC family transporter expression

**Figure 3:**
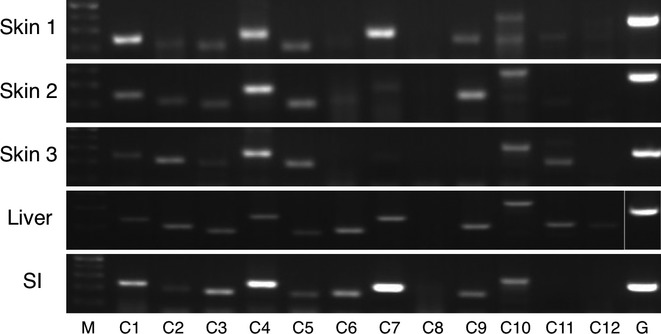
RT-PCR analysis of ABCC family transporter mRNA in human tissues. Total RNA samples of human skin, liver, and small intestine were analyzed by RT-PCR using primers specific for each ABCC subtypes. SI, small intestine; M, 100 base pair marker; G, GAPDH.

It was observed that ABCC4 and ABCC5 were highly or moderately expressed in all of skin samples (Fig. 3). While the higher expression of ABCC1, ABCC7, ABCC9, ABCC10, and ABCC11 was observed in the skin samples, the interindividual variability in the expression level of these subtypes was also shown, as certain individual(s) slightly or barely expressed them in the skin. There were moderate expressions of ABCC2, ABCC3, and ABCC6 in the skin, while ABCC8 and ABCC12 were found not to express in the tissue. In the liver, ABCC family transporters were moderately or slightly expressed except for ABCC8. In the small intestine, ABCC1, ABCC4, and ABCC7 were highly, and ABCC2, ABCC3, ABCC5, ABCC6, ABCC9, and ABCC10 were moderately expressed.

### ABCD, ABCE, ABCF, and ABCG family transporter expression

**Figure 4:**
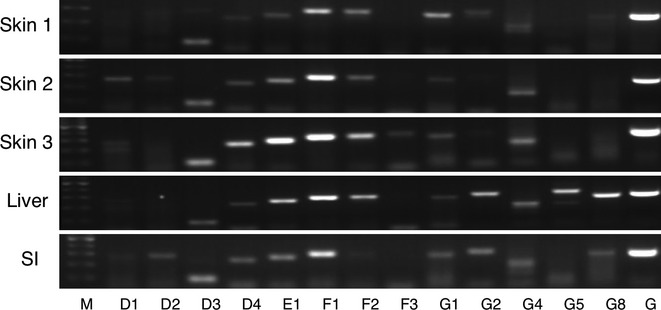
RT-PCR analysis of ABCD, ABCE, ABCF, and ABCG family transporter mRNA in human tissues. Total RNA samples of human skin, liver, and small intestine were analyzed by RT-PCR using primers specific for each ABCD, ABCE, ABCF, and ABCG subtypes. SI, small intestine; M, 100 base pair marker; G, GAPDH.

ABCD3 and ABCD4 were moderately, but equally expressed in all of the skin samples (Fig. 4). ABCD3 was also expressed in the liver and small intestine. ABCD1 and ABCD2 were expressed in the skin; however, there were interindividual variabilities in the expression of these subtypes in the skin. While ABCD2 was expressed in the small intestine, the expression of this subtype was not detected in the liver. ABCD1 and ABCD4 were slightly expressed in the liver and the small intestine.

The ABCE subfamily contains a single member, ABCE1. ABCE1 was highly expressed in the human skin, while one of the skin RNA samples slightly showed its expression (Fig. 4), indicating that there was an interindividual variability in the expression of ABCE1 in the human skin. This subtype was highly and moderately expressed in the liver and small intestine. ABCF1 was highly expressed not only in the human skin but also in the liver and small intestine (Fig. 4), while ABCF2 and ABCF3 were moderately and slightly expressed in these tissues.

Unlike ABCA, ABCB, and ABCC family transporters, the ABCG family members were hardly expressed in the human skin except for ABCG1 and ABCG4, which were moderately expressed in the tested three skin samples (Fig. 4). In the liver, ABCG5 and ABCG8 were moderately, and ABCG1, ABCG2, and ABCG4 were slightly expressed. In the small intestine, all of the ABCG family transporters were slightly expressed except for ABCG5.

### Expression of ABC transporters in normal and cancer skin cells

**Figure 5:**
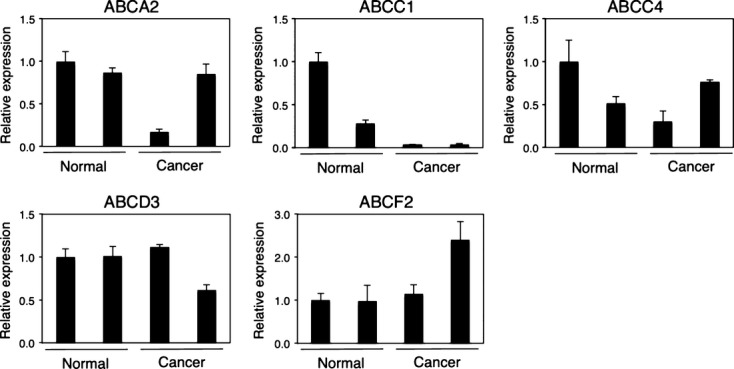
Q-RT-PCR analysis of ABC transporter mRNA in normal and cancer skin. Total RNA samples of human normal and cancer skin (malignant melanoma) were analyzed by RT-PCR using primers specific for ABCA2, ABCC1, ABCC4, ABCD3, and ABCF2. Columns are the mean ± SD of three independent determinations on two separate samples.

We further performed Q-RT-PCR to determine the mRNA expression levels of ABCA2, ABCC1, ABCC4, ABCD3, and ABCF2, which are the ABC transporters expressed in all of the skin samples in our semi-quantitative RT-PCR, in normal and cancer skin. The cycle threshold (Ct) values of ABCA2, ABCC1, ABCC4, ABCD3, and ABCF2 ranged from 22.70 to 31.18, while the average Ct value of GAPDH was 18.23. The expression levels of ABCA2, ABCC4, ABCD3, and ABCF2 in human skin were similar between normal and cancer individuals (Fig. 5). Meanwhile, the level of ABCC1 in skin cancer subjects was eightfold to 26-fold lower than the levels in normal subjects.

## Discussion

Although drugs exhibit their therapeutic effects in their target tissues, they can also cause adverse effects in the body. The skin covers a surface area of approximately 1.7 m^2^ in an average adult body and receives about one third of the circulating blood. Although the skin is the primary tissue that protects our body from ultraviolet irradiation, infection, microorganisms, and exogenous toxic compounds, it is also one of the tissues where severe adverse reactions caused by administered drugs, such as Stevens–Johnson syndrome (Fritsch and Sidoroff 2000), psoriasis (Tsankov et al. 2000), allergy (Kaplan 1984), drug-induced hypersensitivity syndrome (Shiohara et al. 2006), Lyell syndrome (Saiag et al. 1992), and drug-induced photosensitivity (Harber and Baer 1972) are seen. In addition to the adverse effects of drugs themselves, active metabolites formed by metabolism of drugs are suggested to be the causes of drug-induced toxicities (Knowles et al. 2000), as UDP-glucuronosyltransferases (UGTs) catalyze the formation of acyl glucuronide, which is a chemically reactive metabolite that can form covalent adducts with DNA or protein to cause cytotoxicity (Koga et al. 2011). Not only UGTs but cytochrome P450s (CYPs), phase I drug-metabolizing enzymes, have also been reported to form reactive metabolites and be involved in drug-induced toxicities (Yamazaki et al. 1999; Kassahun et al. 2001). Our recent findings and those of others demonstrated that skin cells express a variety of drug-metabolizing enzymes such as CYP and UGT (Baron et al. 2001; Sumida et al. 2013). Taking these data together, it can be speculated that drugs and their reactive metabolites might be the cause of drug-induced toxicities in the skin. Indeed, most drugs that have been reported as causing of drug-related Stevens–Johnson syndrome contain a carboxyl group as a component of their skeletal structures, such as ibuprofen, diclofenac acid, and carbamazepine, which are metabolized to their acyl glucuronides by UGT (Sakaguchi et al. 2004; Staines et al. 2004). Therefore, concentration of drugs in skin cells might be a determining factor in the development of drug-induced cytotoxicity. However, it should be noted that there is significant interindividual variability in the development of these severe adverse reactions to drugs, as the incidence of drug-induced Stevens–Johnson syndrome and psoriasis is particularly low. Given the role of ABC transporters, the expression levels of ABC transporters in the skin would be an important factor in determining the concentration of drugs and metabolites in the skin cells.

Due to the limited availability of primary antibodies that are specific for each ABC transporters, it is unable to determine the protein expression levels of ABC transporters in human tissues. In contrast, mRNA expression levels of ABC transporters can be evaluated by RT-PCR with specific primers. Therefore, the mRNA expression pattern of human ABC transporters in the skin, liver, and small intestine was examined in this study and summarized in Table 2. Since efflux of drugs by ABC transporters in the small intestine and liver plays crucial roles in the absorption and disposition of the drugs, the expression pattern of ABC transporters in these tissues has been widely investigated (Meier et al. 2006; Berggren et al. 2007). ABCC1 (MRP1) and ABCC3 (MRP3) are the members of ABC transporters that are expressed in the small intestine. In agreement with the previous findings (König et al. 1999; Berggren et al. 2007), these subtypes were found to be expressed in the small intestine in this study (Fig. 3). ABCA6, ABCB1, ABCB4, ABCB11, and ABCC6 were reported to express in the liver (Langmann et al. 2003; Meier et al. 2006), which is in accordance with our results.

**Table 2:** Expression profiles of human ABC transporters in skin, liver, and small intestine

Subtypes	Skin 1	Skin 2	Skin 3	Liver	SI
ABCA1		+	+	++	
ABCA2	++	+++	++	++++	++
ABCA3	+			+	
ABCA4				+	
ABCA5	++	++++	+++	++++	+++
ABCA6	+++	++++	+	++++	+++
ABCA7					
ABCA8	+	+		++	++
ABCA9	++	+++	+	+	
ABCA10	+++	++++	+	++	+
ABCA12	+++	+++		+	
ABCA13		+		++	++
ABCB1		+		+	+
ABCB2	++	++		++	+
ABCB3	++	+++	++	++	++
ABCB4	++	+++	+	++++	++
ABCB5		+	+		+
ABCB6	+	+	++++	+++	+
ABCB7	+	+	+++	++	++
ABCB8	+	+	++	+++	
ABCB9	+		+		
ABCB10	++	++	++	++	++
ABCB11	+	+	+	++	
ABCC1	+++	++	+	+	+++
ABCC2	+	+	++	++	+
ABCC3	+	+	+	++	++
ABCC4	+++	+++	+++	++	++++
ABCC5	++	++	++	+	+
ABCC6	+	+		++	++
ABCC7	++++	+	+	++	++++
ABCC8					
ABCC9	+	+++		++	++
ABCC10	+	++	++	++	++
ABCC11	+	+	++	++	
ABCC12				+	
ABCD1		++	+	+	+
ABCD2	+	+			++
ABCD3	++	++	++	++	++
ABCD4	+	++	+++	+	++
ABCE1	+	++	++++	+++	++
ABCF1	+++	++++	++++	+++	+++
ABCF2	++	++	+++	++	+
ABCF3			+		
ABCG1	++	+	+	+	++
ABCG2	+	+	+	++	++
ABCG4	+	++	++	++	++
ABCG5				+++	
ABCG8	+			+++	++

Expression levels of each ABC transporter were displayed by the number of +.

In this study, we identified that various ABC transporters, especially ABCA, ABCB, ABCC, ABCD, ABCE, and ABCF family members, were highly expressed in the human skin. While ABCA5 and ABCF1 were dramatically and equally expressed in all of the skin samples tested in this study, the most significant finding of the present study was that there was significant interindividual variability in the expression levels of ABC transporters in the skin. This would cause the substantial interindividual variability in concentration of drugs and metabolites in the skin cells, especially when the drugs and metabolites are substrates of ABC transporters expressed in the skin. The importance of ABC transporters for cellular concentration of a drug is supported by an in vivo study showing that knockout of ABCC3 caused an increase in a hepatic concentration of acetaminophen, which is probably linking to development of acetaminophen-induced hepatotoxicity (Manautou et al. 2005). To further investigate the potential role of ABC transporters expressed in the skin in the development of drug-induced skin diseases, the function of these ABC transporters, as well as their substrate specificity, needs to be investigated.

Interestingly, we found that the mRNA expression levels of ABCC1 in skin RNA from skin cancer patients were significantly lower compared to the levels in normal subjects (Fig. 5). This indicates that drugs, which are substrates of ABCC1, can accumulate easily in skin cells when the patients develop skin cancer such as malignant melanoma, potentially increasing the risk of developing drug-induced skin diseases. In fact, it has been reported that a patient with stage IV malignant melanoma treated with daily radiotherapy and daily low-dose (100 mg/m^2^) gemcitabine developed Stevens–Johnson syndrome (Sommers et al. 2003). Increased expression of ABC transporters in the skin of the patient might have been the cause of development of the drug-induced Stevens–Johnson syndrome.

Although ABCF1 was highly expressed in the skin (Fig. 4), it would not be directly involved in a transport of the substrates in skin cells, because this subtype lacks trans-membrane domains. Meanwhile, ABCF1 has been identified as a translational regulator of an inflammatory cytokine pathway, as ABCF1 participates in tumor necrosis factor (TNF)-α-induced inflammation (Richard et al. 1998). Therefore, the high expression of ABCF1 in the skin might be involved in the development of drug-induced cytotoxicity by affecting immune responses.

In summary, this is the first study to identify the expression pattern of the whole family of human ABC transporters in the skin. The interindividual variability in the expression levels of ABC transporters in the human skin might be one of the determinants of drug-induced skin diseases. Further investigations are needed to clarify the function of the ABC transporters expressed in the skin, as well as their substrate specificity.

## Abbreviations

ABC transporters: ATP-binding cassette transporters
BSEP: bile salt export pump
CYP: cytochrome P450
GAPDH: glyceraldehyde 3-phosphate dehydrogenase
MDR: multidrug resistance
MRP: multidrug resistance-associated protein
P-gp: permeability glycoprotein
Q-RT-PCR: quantitative reverse transcription-PCR
TAP: antigen peptide transporter
TNF: tumor necrosis factor
UGT: UDP-glucuronosyltransferase
